# Multifunctional Integrated Organic–Inorganic-Metal Hybrid Aerogel for Excellent Thermal Insulation and Electromagnetic Shielding Performance

**DOI:** 10.1007/s40820-024-01409-1

**Published:** 2024-05-23

**Authors:** Zhaoqi Niu, Fengjin Qu, Fang Chen, Xiaoyan Ma, Beixi Chen, Luyao Wang, Miao Xu, Shumeng Wang, Liang Jin, Chengshuang Zhang, Xiao Hou

**Affiliations:** 1 Ministry of Industry and Information Technology Key Laboratory of Special Function and Smart Polymer Materials, Key Laboratory of Materials Physics and Chemistry of Ministry of Education for Extraordinary Conditions, Northwestern Polytechnical University, Xi’an, 710072 People’s Republic of China; 2 Spallation Neutron Source Science Center Institution, Dongguan, 523803 People’s Republic of China; 3 Institute of High Energy Physics, Chinese Academy of Sciences, Beijing, 100049 People’s Republic of China; 4 China Aerospace Science and Industry Corporation Sixth Academy, Hohhot, 022185 People’s Republic of China; 5 Xi’an Aerospace Composites Research Institute, Xi’an, 710025 People’s Republic of China; 6 China Aerospace Science and Technology Corporation, Beijing, 100037 People’s Republic of China

**Keywords:** Multi-hybrid aerogel, Metal–phenolic coordination, Thermal insulation, EMI shielding, Convertibility and multifunctionality

## Abstract

Vehicles operating in space need to withstand extreme thermal and electromagnetic environments in light of the burgeoning of space science and technology. It is imperatively desired to high insulation materials with lightweight and extensive mechanical properties. Herein, a boron–silica–tantalum ternary hybrid phenolic aerogel (BSiTa-PA) with exceptional thermal stability, extensive mechanical strength, low thermal conductivity (49.6 mW m^−1^ K^−1^), and heightened ablative resistance is prepared by an expeditious method. After extremely thermal erosion, the obtained carbon aerogel demonstrates noteworthy electromagnetic interference (EMI) shielding performance with an efficiency of 31.6 dB, accompanied by notable loading property with specific modulus of 272.8 kN·m kg^−1^. This novel design concept has laid the foundation for the development of insulation materials in more complex extreme environments.

**Supplementary Information:**

The online version contains supplementary material available at 10.1007/s40820-024-01409-1.

## Highlights

- The homogeneous hybridization of organic–inorganic-metal elements in 3D aerogel was achieved by an expeditious method.
- The aerogel tightly integrates excellent thermal insulation (49.6 mW m^−1^ K^−1^), ablative resistance, mechanical strength, and superhydrophobic properties.
- The ablated carbon aerogel combines notable electromagnetic interference shielding properties (31.6 dB) and load-carrying properties (272.8 kN·m kg^−1^).

**Supplementary Information:**

The online version contains supplementary material available at 10.1007/s40820-024-01409-1.

## Graphical abstract

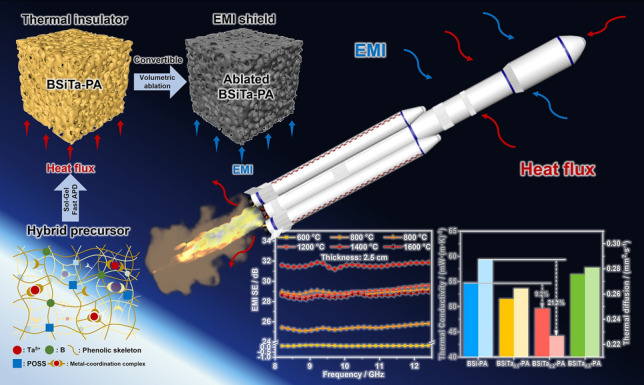

## Introduction

The expanding demands of launch vehicles have promoted the booming development of high-performance lightweight functional materials [1–3]. Aerogels, due to its exceptional low density and insulating capabilities, are in expanding demands in the field of space vehicles [4–8]. In the practical applications, space vehicles not only need to withstand the extreme heat erosion, but also need to block electromagnetic interference from cosmic space [8–12]. However, due to separate performance-oriented material design, there are few reports of compatibility and integration of these two features.

As a representative of organic aerogel, phenolic aerogel possesses excellent thermal stability and high-temperature shape retention rate upon traditional organic aerogel, which has been applied in the field of aerospace thermal insulation [13–15]. For thermal protection-oriented aerogels, serious carbonization and ceramization usually occur during the extremely thermal protection process [16]. Coincidentally, for EMI shielding aerogels, their properties are affected by the material’s intrinsic chemical structure, porous structure, degree of graphitization, and electrical conductivity [17]. Accordingly, to fit the advancement of multifunctional aerospace materials, an interesting strategy is proposed to develop a convertible aerogel that can transform into an EMI shield after undergoing extreme thermal environmental ablation, which will not only enable the integration of performance, but also the full utilization of the material. For the convertible strategy, the carbonization capacity is crucial for its convertible performance. Studies have shown that covalently bonded inorganic and metal hybrid elements have positive effects on the thermal stability and ablative resistance performance improvement of phenolic resin, because the hybrid elements endow a more stable chemical structure and can delay the pyrolysis process of the resin with forming carbon/ceramic structure to withstand the heat flow erosion [18, 19].

Recently, the reported modified phenolic aerogels usually comprise only one or two functional elements, lacking the further integration and development of functional features [20, 21]. The hybrid phenolic precursor containing three or more functional elements and its three-dimensional aerogel, however, has not been reported, owing to the difficulties for material preparation: (i) The utilization of modifiers needs to consider not only the amount and type of reactive sites in the chemical structure of the phenolic resin to meet the requirements of the chemical reaction during the modification process, but also the reaction between the modifier and the phenolic resin, which will affect its molding process and material properties; (ii) hybrid phenolic resin precursor solutions exhibit strong intramolecular and intermolecular non-covalent interactions, especially π stacking, hydrogen bonding and coordination, leading to a rapid phase separation and co-precipitation of hybrid phenolic sol rather than the formation of a uniform gel network due to this unstable sol–gel process. Therefore, the uniform integration of multiple hybrid elements as inorganic and metal components into 3D high-performance hybrid aerogels is a major challenge.

Here, we report the development of organic–inorganic-metal hybrid aerogel with convertible features and multifunctional properties. The hybrid aerogels are obtained by sol–gel preparation of Ta^5+^-chelated boron–silicon phenolic resin (catalyzed by polyhedral oligomeric silsesquioxanes (POSS)), followed by rapid ambient pressure drying (APD) process. Tantalum has excellent high temperature resistance, and the TaC formed by high-temperature ablation possesses both excellent electrical conductivity and high melting point, which not only is a key for its thermal insulation, but also the guarantee to its EMI shielding efficiency. Thanks to the performance gains by selected functional elements, the resulting hybrid aerogel exhibits low density, excellent thermal stability, mechanical strength, thermal insulation, and ablative resistance. After volumetric ablation, the hybrid aerogel turns to be a carbon aerogel, possessing good ceramization, electrical conductivity, and multi-scale micro-/nanopore structure, presenting a good EMI performance. To deeply understand the structure–performance relationship of the hybrid aerogel, we investigated the microstructure evolution of the hybrid aerogel pyrolyzed at different ablated temperatures. The crystal structure evolution of carbon and ceramic was analyzed by X-ray diffraction (XRD), X-ray photoelectron spectroscopy (XPS), and Raman spectroscopy. Meanwhile, small-angle X-ray scattering (SAXS) and mercury intrusion porosimetry (MIP) were adopted to analyze the nano-micron pore structure of the ablated carbon aerogel. This work demonstrates the feasibility of the proposed design concept and the great potential of multicomponent hybrid aerogel as a lightweight and promising multifunctional material.

## Experimental Section

### Materials

The *p*-toluenesulfonic acid, ethanol–tantalum, and anhydrous ethanol were purchased from Aladdin Biochemical Technology Co., Ltd. (Shanghai, China). The boron–silica hybrid phenolic resin catalyzed by POSS was synthesized according our previously report [18]. All chemicals were used without further purification.

### Preparation of Boron–Silica–Tantalum Hybrid Phenolic Aerogel and Carbon Aerogel

**Fig. 1:**
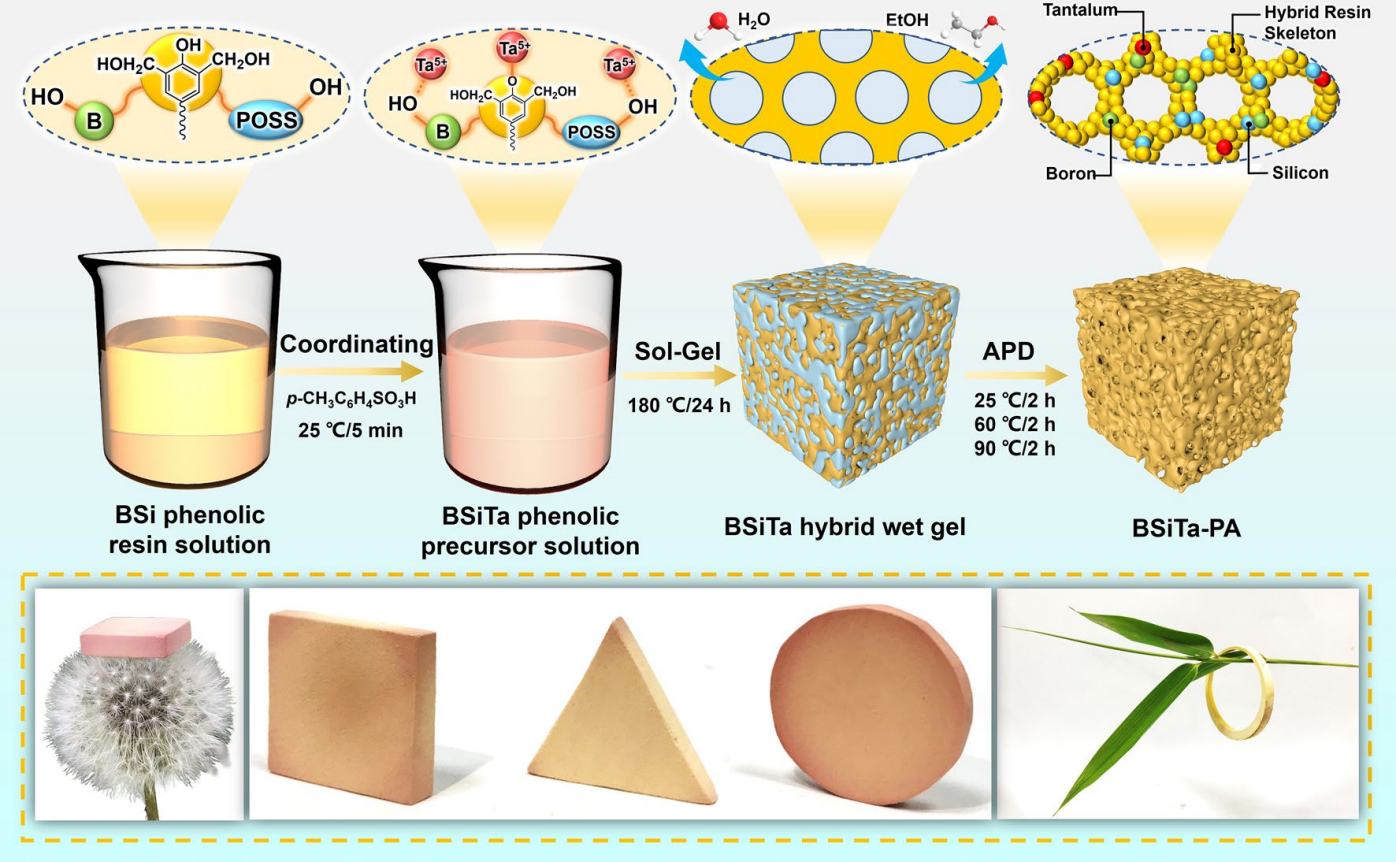
Schematic description of the multifunctional aerogel preparation process and photographs (bottom) of the aerogel with different geometric shapes

The entire preparation procedure of the boron–silicon–tantalum ternary hybrid phenolic aerogel (BSiTa-PA) is shown in Fig. 1. The boron–silica–tantalum hybrid phenolic aerogel was prepared by covalent and coordination hybridization, accompanied by sol–gel and fast APD process. Specifically, 15 g of the boron–silica hybrid phenolic resin was dissolved in 80 g of anhydrous ethanol to obtain a 15.8 wt% resin solution. Afterward, 0.03 g of ethanol–tantalum was added to 20 g of anhydrous ethanol and stirred to obtain a homogeneous solution. The resin solution of 15.8 wt% was slowly added to the above ethanol–tantalum ethanol solution and stirred evenly to obtain the boron–silica–tantalum homogeneous hybrid resin precursor solution. During the coordination process, Ta(OC_2_H_5_)_5_ rapidly initiated self-assembly with the hybrid phenolic resin, upon diffusion into the boron–silica hybrid phenolic resin solution. Then, 0.75 g of *p*-toluenesulfonic acid was added into the boron–silica–tantalum hybrid resin precursor solution as a catalyst and stirred evenly. Finally, the hybrid aerogel was prepared by a simple high-pressure assisted polymerization combined with fast APD process. The aging process of sol–gel and gel was completed by placing the resin solution in an autoclave at 180 °C for 24 h. Once the ambient temperature reached the polycondensation temperature of hybrid precursor, the hybrid precursor promptly cross-linked and separated from the solvent phase to form a uniform hybrid wet gel, containing B, Si, and Ta. The gel was removed without solvent exchange and dried at atmospheric pressure for 6 h to replace the solvent and air inside the gel. The product was named BSiTa_0.2_-PA. Other samples were prepared by changing the concentration of ethanol–tantalum. The concentration of the precursors (resin and *p*-toluenesulfonic acid) remains constant and is indicated by the ratio of the weight of the precursors to the weight of the sol. The hybrid aerogel without tantalum was named BSi-PA, and the tantalum hybrid aerogel was named BSiTa_*x*_-PA (*x* = 0.1, 0.2, 0.3, where* x* indicates the trend of the amount of ethanol-tantalum).

BSiTa_0.2_-PA was placed in a tube furnace protected by Ar atmosphere and heated at a rate of 10 °C min^−1^ to 600, 800, 1000, 1200, 1400, and 1600 °C, respectively, and kept at each temperature point for 1 h. After cooling to room temperature at the same rate, the aerogels produced by volumetric ablation at different temperatures can be obtained.

## Results and Discussion

### Preparation and Microstructure of BSiTa-PA

The Fourier transform infrared spectrometer spectra of the as-prepared B, Si, and Ta hybrid resin and the B, Si hybrid resin without Ta^5+^ as reference are displayed in Fig. S1a. It is evident that Ta(OC_2_H_5_)_5_ addition greatly lowers the hydroxyl peak and the peak shifts from 3253 to 3307 cm^−1^, indicating that Ta^5+^ is coordinated to form a stable chelated structure [22]. In addition, the results of thermogravimetric analysis (TGA) and derivative thermogravimetric (DTG) curves of the cured hybrid resins in Fig. S2b, c show that the hybrid resins possess advanced thermal stability, and the pyrolysis trend of the BSiTa_0.2_ hybrid resin in the initial decomposition stage (< 600 °C) is completely different from that of the BSi hybrid resin, with the thermal decomposition significantly delayed. At this stage, the reason of decomposition is mainly due to the dehydration of hydroxyl groups, etc., and thus this also indirectly proves the successful coordination of Ta^5+^. The chemical structure analysis of BSiTa_0.2_-PA by XPS is shown in Fig. S2d–f, in which B, Si and Ta^5+^ are all bonded to the organic skeleton of phenolic aerogel through covalent and coordination bonds. The BSiTa_*x*_-PA hybrid aerogel monoliths are amenable to subtractive manufacturing processes, enabling the creation of a variety of desired shapes, including cuboids, tri-prisms, cylinders, and rings.

**Fig. 2:**
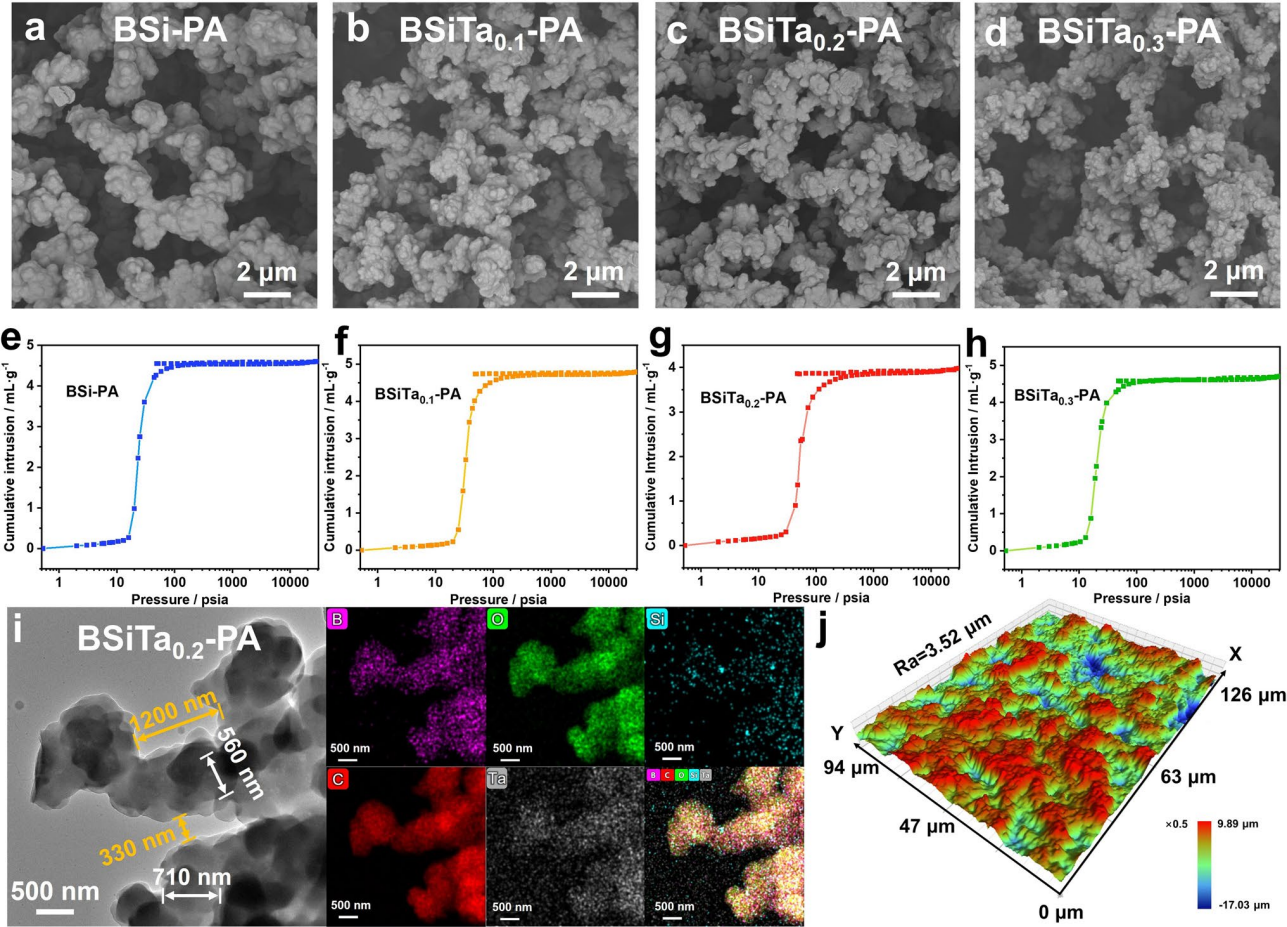
Morphological and structural characteristics of BSi-PA and BSiTa-PA aerogels. **a-d** SEM images of fracture surface of the aerogels with different Ta^5+^ contents, **e–h** intrusion and extrusion curves of mercury and the attaching pore size distributions. **i** HRTEM and elemental mapping images show the nanostructure and the homogeneous distribution of the hybrid element domain. **j** Laser optical 3D surface image of the BSiTa_0.2_-PA

The microscopic morphological characteristics of the prepared phenolic aerogels are presented in Fig. 2. The scanning electron microscope (SEM) images showed that the aerogel network was composed of primary nanoparticles and submicron clusters, and all aerogels showed similar three-dimensional thick neck micro-nano-network structure, which was different from the traditional thin neck "pearl necklace" network structure of inorganic aerogels, which may indirectly indicate that the aerogel possesses better mechanical strength (Fig. 2a–d); meanwhile, according to the Young–Laplace equation (Eq. S1), the existence of macro-scale pores observed from SEM images is one of the necessary conditions for successful APD process, which can effectively reduce the capillary force and avoid the collapse of the gel structure [6].

Since there are many macro-scale pores in aerogel, the MIP was used to further analyze the microscopic features of the pores, as shown in Fig. 2e–h, S4 and Table S2. With the addition of Ta(OC_2_H_5_)_5_, the secondary particles with the size of tens of nanometers tend to form finer microstructure, while the specific surface area and average pore size of macro-scale pores of aerogel are also affected by the Ta^5+^ content. The results show that the pore specific surface area is 33.3–44.2 m^2^ g^−1^, of which the maximum value is 44.2 m^2^ g^−1^ of BSiTa_0.2_-PA, and the average pore diameter of macroscopic scale is 2.4–6.7 μm, of which the minimum is 2.4 μm of BSiTa_0.2_-PA. This transformation may be due to the rapid self-assembly between hybrid phenolic precursor and Ta^5+^ with appropriate content to form more stable chelated macromolecules.

Therefore, as a representative, the microstructure of the BSiTa_0.2_-PA aerogel hybrid domain was further examined by high-resolution transmission electron microscopy (HRTEM) and energy-dispersive X-ray spectroscopy (EDS) element mapping shown in Fig. 2i. It was found that the hybrid skeleton structure composed by organic phenolic resin and boron, silicon, and tantalum hybrid elements have continuous amorphous phases. The element mapping images confirmed the uniform distribution of silicon, boron, and tantalum components in the aerogel skeleton, which was attributed to the ethanol-stabilized hybrid phenolic heat-induced sol–gel rapid phase separation process.

The surface morphology of hybrid aerogel was studied by laser optical 3D imaging, as shown in Figs. 2j and S3. The roughness of BSi-PA aerogel is 2.88 μm, whereas it is 3.52 μm in BSiTa_0.2_-PA aerogel. The increase of surface roughness may significantly improve the hydrophobic properties of the aerogel, as is shown in Supporting Information.

### Thermal Stability, Mechanical Property, and Thermal Insulation Performance of BSiTa-PA

**Fig. 3:**
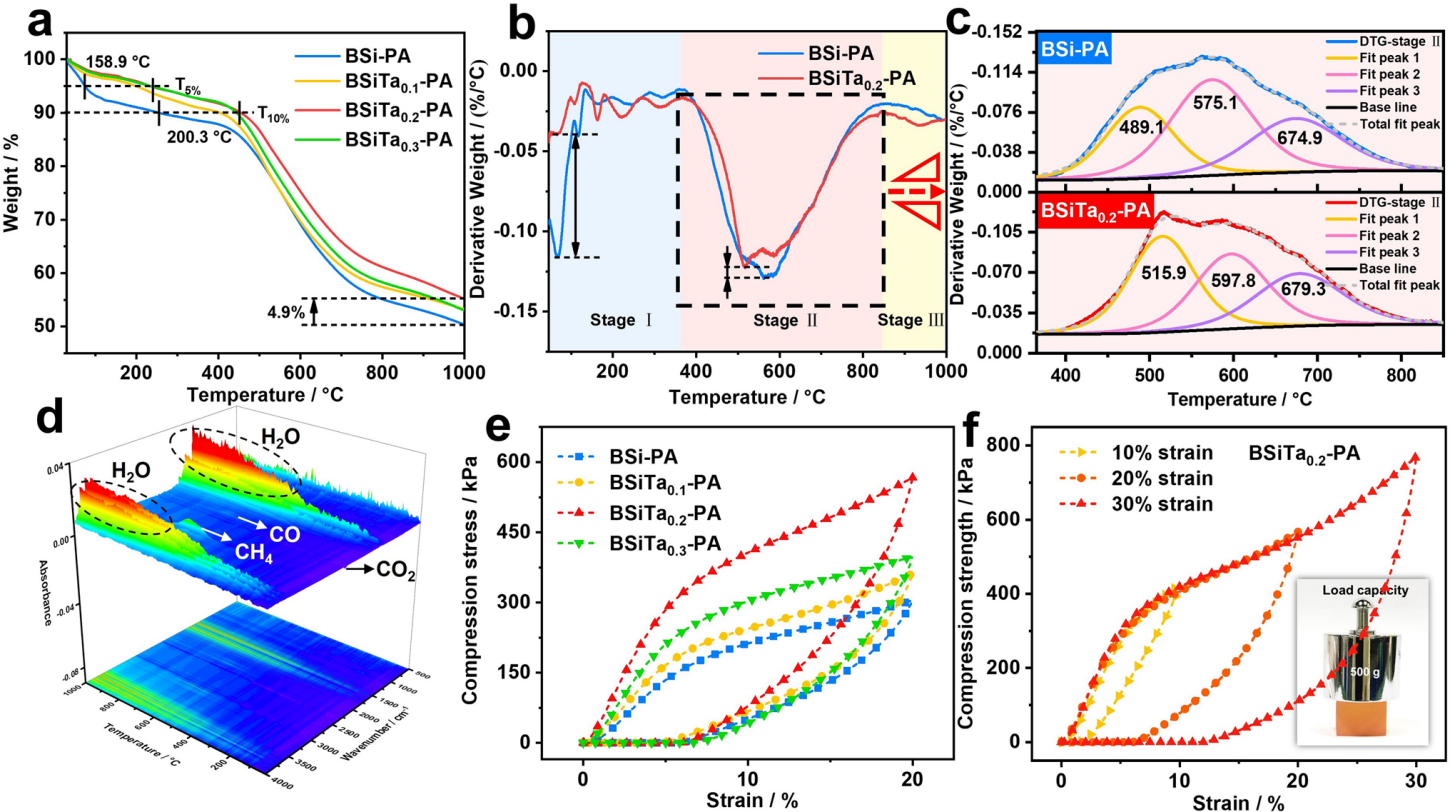
Thermal stability properties: **a-b** TGA and DTG curves of hybrid aerogels,** c** the deconvoluted peaks of DTG stage II, **d** three-dimensional mapping TG-IR diagram of volatile product for BSiTa_0.2_-PA. Mechanical properties: **e** *σ*−*ε* curves of BSi-PA and BSiTa-PA aerogels under 20% *ε*, **f** *σ*−*ε* curves of BSiTa_0.2_-PA aerogels with increased compressive *ε* and intuitive representation of the mechanical property

As an advanced thermal protection material, aerogel's thermal insulation and ablative resistance performances are of vital importance [23–26]. Specifically, thermal stability is a significant factor for ablative resistance [27–31]. As shown in Fig. 3a, with the increase of Ta^5+^ content, the thermal stability of the aerogels displays the tendency of rising up at the beginning and then declining in late. The initial decomposition temperature (*T*_5%_ and *T*_10%_, representing the temperature corresponding to 5 and 10 wt% decomposition) of BSiTa_0.2_-PA is 158.9 and 200.3 °C higher than those of BSi-PA, respectively, and the char yield at 1000 °C of BSiTa_0.2_-PA is 4.5% higher than that of BSi-PA, indicating the aerogel with appropriate Ta^5+^ possess better thermal stability. The thermal stability of BSiTa_0.2_-PA has significant advantages over the reported high-performance aerogels, as shown in Table S5. Meanwhile, according to DTG curves, the pyrolysis of aerogels can be roughly divided into three stages (Fig. 3b). In the first stage, the degradation rate of BSiTa_0.2_-PA is significantly lower than that of BSi-PA. In the second stage, the degradation rate of BSiTa_0.2_-PA is also slightly lower than that of the sample without Ta. Since the second stage is the main pyrolysis stage, this stage is deconvolution and roughly divided into three pyrolysis peaks, as presented in Fig. 3c. It can be seen that the three pyrolysis peaks of BSiTa_0.2_-PA are all higher than those of BSi-PA. It is possible because that the appropriate amount of Ta^5+^ coordination introduces Ta–O bridge into the cross-linking network of phenolic resin, where the Ta–O bond energy is up to 849 kJ mol^−1^ [32], much higher than the C–O and C–C chemical bonding energies. Furthermore, the composition analysis of the gas release of BSiTa_0.2_-PA sample upon increasing temperature was conducted to understand the pyrolysis process by thermogravimetric infrared (TG-IR) in Fig. 3d. It can be seen that the release of H_2_O is mainly accompanied by the release of a few small carbon-containing molecules, such as CO_2_, CO, and CH_4_.

These results can be attributed to the following three reasons: (i) The solvothermal preparation process leads to the existence of a large number of unreacted end-hydroxyl group in the phenolic aerogel, which releases a large number of water molecules during the pyrolysis process; (ii) the cross-linking structure of BSiTa_0.2_-PA hybrid phenolic aerogel is complex, in which the degree of cross-linking is large, and the relative content of the chain end segment in the cross-linking network is small, and thus, the initial decomposition temperature is higher; (iii) the hybrid elements, B, Si, Ta, and other hybrid elements, have strong "carbon fixation" ability and can undergo ceramic reaction with pyrolytic carbon in the heating process, and therefore, the char yield of BSiTa_0.2_-PA is higher.

For thermal protection application, excellent mechanical robustness serves as a crucial assurance for aerogel to overcome deformation in extreme environments and facilitates the arbitrary shape preparation and subtractive manufacturing [33–35]. The compression performance of BSiTa-PA was tested and compared with the control sample (BSi-PA). The typical compressive stress–strain (*σ* − *ε*) curves and modulus are shown in Fig. 3e, f and Table S2, in which BSiTa_0.2_-PA shows the highest strength and modulus. Based on the classical deformation response of porous materials, the upward convex ring can be divided into three stages. In the first stage (the linear elastic deformation of BSiTa_0.2_-PA (*ε* < 5%)), the energy loss is relatively low with the corresponding less irreversible deformation, in which the slope of the stress–strain curve is used to derive Young's modulus. Next, after reaching the yield point, plastic deformation such as fracture of aerogels partial skeleton and joint failure occurs gradually. At last, the entire aerogel is densified.

To further demonstrate the compression flexibility of isotropic BSiTa_0.2_-PA, uniaxial loading and unloading experiments with different strains were carried out, whose results are shown in Fig. 3f. It shows that as Ta^5+^ content increases, the compressive strength and modulus of the sample also display the tendency of rising up at the beginning then declining in late, in which the peak values occur in the case of BSiTa_0.2_-PA sample, with the compressive strength (*ε* = 30%) of 768.7 kPa and modulus of 7.1 MPa, respectively. The possible reason would be that Ta^5+^ can form a stable chelated structure with phenolic organic structures, whose Ta–O bond energy is relatively higher [32]. However, with the excessive addition of Ta^5+^, the mechanical properties decrease, indicating the stable chelated structure is disrupted. Since BSiTa_0.2_-PA aerogel has the best mechanical strength and abundant micro-nanostructure, BSiTa_0.2_-PA is chosen as the target materials in this work for the following performance investigation and microstructure analysis.

The thermal protection performance of the hybrid aerogels was assessed through static thermal insulation and dynamic ablative thermal insulation measurements, accompanied by a detailed analysis of the underlying mechanism via finite element method (FEM).

**Fig. 4:**
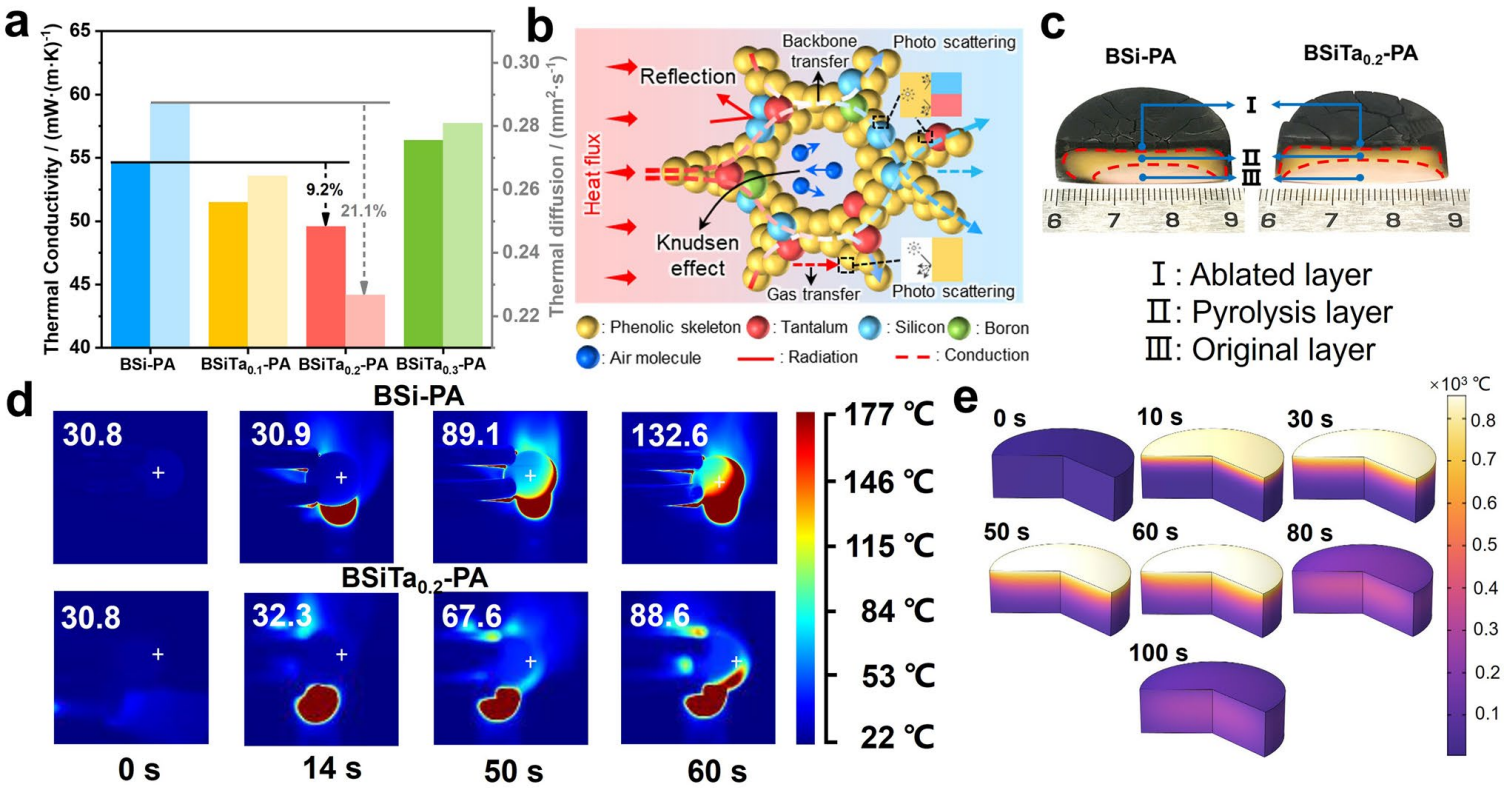
Thermal insulation properties: **a** thermal conductivity and thermal diffusion of hybrid aerogels, **b** schematic illustration of the heat transfer process of the BSiTa_0.2_-PA, **c** ablation cross sections of BSi-PA and BSiTa_0.2_-PA, **d** infrared thermographic images of the BSi-PA and BSiTa_0.2_-PA back side,** e** temperature field simulation cloud image of BSiTa_0.2_-PA (0–60 s for ablation, 60–100 s for natural cooling)

Firstly, the thermal conductivity of hybrid aerogel was investigated, as shown in Fig. 4a. Theoretically, the thermal conductivity of aerogel in air (*λ*_total_) can be expressed as follows [36]:

1 $${\lambda }_{{\text{total}}}={\lambda }_{{\text{conv}}}+{\lambda }_{{\text{rad}}}+{\lambda }_{{\text{air}}}+{\lambda }_{{\text{solid}}}$$

where *λ*_conv_ represents heat convection; *λ*_rad_ is the contribution of thermal radiation; *λ*_air_ and *λ*_solid_ are thermal conductivity of gas phase and solid phase, respectively. Here, *λ*_conv_ should be insignificant because the micro-/nanopore size of the prepared aerogels is much lower than the starting size of natural convection (> 1 mm); *λ*_rad_ should also be ignored because the in BSiTa_0.2_-PA not only the infinite pore walls in the aerogel act as a heat shield, but also the silica in the skeleton is a highly effective anti-radiation material. In this case, therefore, the thermal conductivity of the hybrid aerogel is only related to *λ*_solid_ and *λ*_air_. But since *λ*_air_ is suppressed and relatively stable due to the "Knudsen effect", *λ*_solid_ has the decisive effect on *λ*_total_. The thermal conductivity property of the aerogels at room temperature shows that BSiTa_0.2_-PA exhibits better thermal insulation features than others at the same bulk density, with thermal conductivity (*λ*) and thermal diffusion coefficient (*α*) as low as 49.6 mW m^−1^ K^−1^ and 0.22 mm^2^ s^−1^ (Fig. 4a). The thermal insulation mechanism is shown in Fig. 4b. This good performance can be ascribed to the abundant multi-scale micro-/nanopore structure, including the intrinsic pores of the aerogel and the cage structure of POSS in its skeleton, low thermal conductivity of Ta–O and Si–O bond as excellent phonon barriers, greatly reducing *λ*_solid_ conduction. Additionally, due to the excellent thermal insulation and mechanical elasticity of BSiTa_0.2_-PA, infrared stealth properties can be endowed as a bonus (Fig. S7c). For a more intuitively observation of this difference between static thermal insulation performances, the thermal insulation performance of the hybrid aerogels was tested on a static heat source at a constant temperature (130 °C) in Fig. S7a. The result shows that within 5 min, the surface temperature of BSiTa_0.2_-PA always maintains a lower temperature compared to that of BSi-PA.

Furthermore, the butane torch flame was used to examine the dynamic ablative thermal insulation performance of hybrid aerogel. The variation of its back temperature was measured by infrared thermal imager, as shown in Fig. S7d. According to the pyrolysis state of the aerogel, the ablation cross section of the aerogel in Fig. 4c can be divided into three regions: (i) ablated layer, (ii) pyrolysis layer, and (iii) original layer. It can be intuitively seen that BSiTa_0.2_-PA obviously retains more original layer. In Fig. 4d, after the ablation for 60 s, the central back temperature of BSiTa_0.2_-PA is always lower than 90 °C, while the central back temperature of BSi-PA at 60 s has reached 132.6 °C. The reasons for this excellent thermal protection performance can be attributed to the following two aspects: (i) in the pyrolysis/ablation process of hybrid aerogel, the hybrid elements can undergo complex ceramic reactions, and the formed ceramic structure can act as an effective thermal barrier to resist further erosion by heat flow; (ii) due to the existence of POSS and metal–organic linkage (Ta–O) with low thermal conductivity, proper content of POSS and Ta–O structure can effectively improve the thermal insulation performance of bulk aerogel.

Herein, a three-dimensional ablative model of hybrid aerogel was established by FEM, considering the mechanism of heat transfer/heat dissipation such as surface heat erosion, heat radiation and heat convection. Through theoretical simulation and calculation, the temperature field cloud image and back temperature variation rule of BSiTa_0.2_-PA sample during the ablation process were obtained, as shown in Figs. 4e and S8, S9. Based on the comparison between the experimental data and the simulation results, it can be seen that the peak back temperature obtained by the FEM simulation is similar to the experimental results, and the simulated back temperature curve is basically consistent with the fitting curve of the experimental back temperature, so the theoretical calculation results are convincing. Thus, we can accurately obtain the temperature changes and location of the ablative surface, pyrolytic layer, and original material of the sample from the obtained temperature field, combined with the thermal stability features of the sample, so as to conduct subsequent microstructure analysis.

### Ceramic/Carbon Structural Transformation and Efficient EMI Shielding Properties of Multi-Hybrid Carbon Aerogel

Ablation of thermal protection is usually divided into surface ablation and volumetric ablation, representing the removal of materials from heat-exposed surfaces and the decomposition of internal materials, respectively [37]. As a result, the portion that undergoes volumetric ablation internally usually forms a bulk carbon material, which maintains its structural integrity and confers additional functional properties. As mentioned in Sect. 3.2, in addition to the excellent thermal stability, the high aromatic ring packing density makes phenolic also have an excellent high-temperature shape retention, which is the reason that phenolic is often used as a precursor to carbon materials, especially hard carbon [13, 38].

**Fig. 5:**
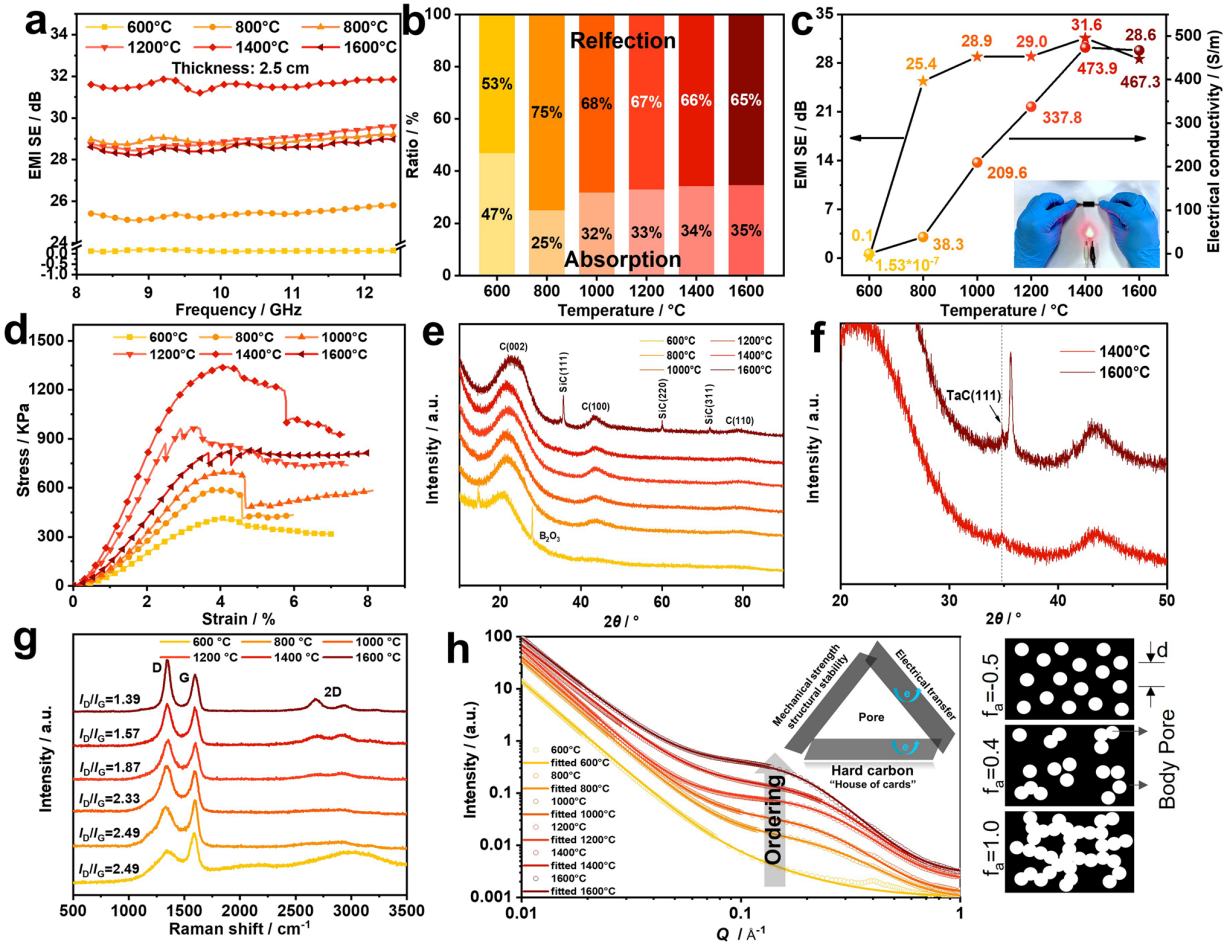
EMI shielding and electrical conductivity performance: **a** EMI SE–Frequency curves for aerogels under different ablation temperature, **b** the absorption and reflection ratio of EMI SE, **c** average EMI SE and electrical conductivity of aerogels. Mechanical property: **d** compression *σ*−*ε* curves of ablated aerogel. Ceramic/carbon/pore structure transformation analysis of different volumetric ablated aerogel: **e, f** XRD, **g** Raman spectra, **h** SAXS

Through volumetric ablation of BSiTa_0.2_-PA in the gradient temperature range, the EMI shielding performance of its ablated aerogel monolith with different levels of carbonization/ceramization in the X-band (8.2–12.4 GHz) was analyzed (Fig. 5a–c and Table S4). As the ablation temperature increases, the absorption and reflection losses are redistributed. When the ambient temperature reaches 1400 °C, the obtained aerogel has the best EMI shielding capability with an average efficiency of 31.6 dB, which can shield 99.9% of electromagnetic interference. By combining the TGA and electrical conductivity characteristics of BSiTa_0.2_-PA, it can be seen that under a temperature of 600 °C, the sample has not been completely pyrolyzed; therefore, the existence of organic structure makes its electrical resistance larger, with an electrical conductivity of only 1.53 × 10^–7^ S m^−1^. When the temperature is higher than 800 °C, the char yield of the sample gradually tends to be stable, accompanied with the electrical conductivity increases exponentially (reaches 473.9 S m^−1^ at 1400 °C), which indicates that the carbon component in the aerogel forms a well-conductive structure. For stably practical application in EMI shielding, the mechanical strength of aerogel is crucial [39]. As shown in Fig. 5d and Table S3, with the increasing of ablation temperature, the skeleton strength of hybrid carbon aerogel exhibits the tendency of rising up at the beginning then declining in late, in which the peak specific modulus reaches 267.4 kN·m kg^−1^ at 1400 °C. When the heat treatment temperature further increases to 1600 °C, the specific modulus decreases to 147.7 kN·m kg^−1^.

To explain the fine EMI shielding, ablative resistance and mechanical robustness of the obtained aerogel by high-temperature volumetric ablation, the microstructure of the aerogel at different temperatures was analyzed by XRD, SAXS, XPS, Raman spectroscopy, MIP, and SEM. Figure 5e, f shows the XRD of BSiTa_0.2_-PA samples pyrolyzed in the range of 600–1600 °C. It can be seen that these dominant peaks ascribed to (002), (100), and (110) reflections of graphite-like structure (with space group of *P*6_3_/*mmc*) are obviously broad, which may be due to the small domain of graphite microcrystals and the existence of defects. With the increase of ablation temperature, these peaks turn to be shaper, in which the (002) peak gradually moves to a higher 2*θ* angle, indicating that the crystallinity of the sample is increasing (although the entire crystallinity level is low compared with graphite), with a decreasing interlay distance of carbon layers [40]. Meanwhile, sharp Bragg peaks appear in at 1600 °C, indexing to (111), (220), and (311) reflections from SiC (*F*_34*m* space group). An additional small fraction of sharp peak was observed at 2*θ* angle of around 34.8°, associated with a small amount of TaC ceramic crystal as shown in the enlarged region in Fig. 5f. Under 600 °C, two peaks at 14.7° and 27.9° appear, ascribed to B_2_O_3_. The structural evolution at various temperature is corroborated by the analysis of XPS and Gibbs free energy of thermochemical reactions (Fig. S10a–d).

According to the Raman spectra (Fig. 5g), the graphitization degree (*I*_D_/*I*_G_) of the carbon layer structure increases with the increasing temperature. When the ablation temperature reaches 1400 °C, the value of *I*_D_/*I*_G_ goes to 1.57, and the 2D peak at 2500–3000 cm^−1^ is gradually remarkable, demonstrating the formation of the graphite structure. Since graphite microcrystals are effective carriers of electron transport, the degree of graphitization is positively correlated with the electrical conductivity [41]. Meanwhile, highly conductive TaC crystal appears at 1400 °C, further improving the electrical conductivity [42]. The increase in conductivity gives rise to a strong impedance mismatch, thus leading to more reflection losses [43]. In addition, with the increase of ablation temperature, the ceramic structure of aerogel gradually becomes stable, in which a large amount of heterogeneous interfaces between the graphite and SiOC amorphous composite ceramic, etc., formed at 1400 °C can produce a certain polarization loss and contribute to the EMI shielding performance [44].

The multiple reflections caused by the multi-scale pore structure in the aerogel can effectively enhance the interface polarization, to improve the absorption ability of electromagnetic interference [39, 45]. In Fig. S10e, f and Table S3, the MIP results show that with the increase of the volumetric ablation temperature, the average macropore reaches the minimum value of 1291.6 nm when it reaches 1400 °C. In that case, the EMI shielding performance also peaks compared to cases at other temperature points. However, when the temperature further increases to 1600 °C, the average diameter of macropores increases, which may be because the Si-based ceramic vaporizes at this temperature point and overflow from the aerogel skeleton, generating part of the gas pore and resulting in the further expanding of the existing large pore.

Phenolic resins are usually an important source of hard carbon because of their inability to form fully cross-linked precursors in a semi-fluid state during pyrolysis [38]. This microstructure of hard carbon is a non-graphitic carbon material with tiny small (< 20 Å) graphite domains inside [46]. It is supposed to form many nanopores in the materials surrounded by small graphite domains (as shown in Fig. 5h) [47]. However, MIP and SEM are not adequate to evaluate the nano-scale pores especially the closed nanopores, and thereby SAXS has been performed to identify the nanopores including closed pores [48]. The details about theoretical models used for refining the SAXS intensity are presented in Supporting Information, to extract the pore size distribution and volume fraction of pores. The applied Teubner–Strey model gives the information of an average distance *d* between pores, the amphiphilic factor *f*_*a*_ as a disorder parameter and the average pore radius *r* [49]. The SAXS fitting results listed in Figs. 5h and S11 show that as increasing the ablation temperature (800–1600 °C), the pores feature of samples displays corresponding variations. The average pore radius *r* is less than 10 Å, confirming the nano-scale porous character in volumetric ablative samples, which turns to be smaller (from 8.64 to 4.63 Å) with the increasing temperature. The disorder parameter *f*_*a*_ decreases from ~ 0.72 to ~ 0.07 (at 1000–1600 °C), which is smaller than that for randomly spread globule shaped pores (*f*_*a*_ = 0.4), representing an increasing degree of short-range pore–pore ordering. In particular, the pore–pore distance *d* increases with the temperature from 1000 to 1600 °C and, however, is slightly higher in the sample at 800 °C. This exception is possibly due to the less-concentrated large pores with random distribution in the sample processed at 800 °C, which is consistent with its largest *d*, *r* and *f*_*a*_ values.

Based on the above microstructure analysis, we can draw a safe conclusion that the aerogel after ablation can turn to be a carbon material with similar structure of hard carbon, which possesses a turbostratic structure (so called “house of cards”) showing great advantages in mechanical strength and structural stability, and also make a contribution to the EMI shielding. With the further increase of temperature, some gas pores are generated due to the overflow of some hybrid element in aerogel, which leads to the weakening of skeleton strength. Although the degree of graphitization is further improved at 1600 °C, its compression modulus is still not stable at this stage.

## Conclusions

In summary, to satisfy the more extreme complex thermal and electromagnetic environments in cosmic space, a new boron–silicon–tantalum ternary hybrid phenolic aerogel (BSiTa-PA) with excellent machinability, mechanical properties (39.4 kN·m kg^−1^), superhydrophobic (> 150°), thermal insulation (49.6 mW m^−1^ K^−1^), and ablative resistance properties was prepared. After extremely thermal erosion, the obtained carbon aerogel monolith demonstrates noteworthy EMI shielding performance with an efficiency of 31.6 dB at 1400 °C, accompanied by good load-carrying property with specific modulus of 272.8 kN·m kg^−1^. An in-depth understanding of the microstructural evolution of the aerogel during volumetric ablation was found that the small graphite domains, good ceramic structures, and abundant nanopores during the ablation process, in conjunction with the pore structure inherent to aerogel, are the key reason for the excellent ablative resistance and electromagnetic shielding properties of the aerogel. The development of this convertible, multifunctional aerogel lays the foundation for the advancement of insulating materials in next-generation aerospace applications.

## Supplementary Information

Below is the link to the electronic supplementary material.

Supplementary file 1 (PDF 1171 KB)
