# Supplementary material for: Multifunctional Integrated Organic–Inorganic-Metal Hybrid Aerogel for Excellent Thermal Insulation and Electromagnetic Shielding Performance
Source: Nanomicro Lett. 2024 May 23;16:200. doi: 10.1007/s40820-024-01409-1 (PMC11116317; doi:10.1007/s40820-024-01409-1)
Supplement: Supplementary file 1 — (PDF 1171 KB) [file 40820_2024_1409_MOESM1_ESM.pdf]

Supporting Information for

Multifunctional Integrated Organic-Inorganic-Metal Hybrid Aerogel for Excellent Thermal Insulation and Electromagnetic Shielding Performance

Zhaoqi Niu[1], Fengjin Qu[2, 3], Fang Chen[1], Xiaoyan Ma[1, *], Beixi Chen[1], Luyao Wang[1], Miao Xu[1], Shumeng Wang[1], Liang Jin[4], Chengshuang Zhang[5] and Xiao Hou[1, 6, *]

[1] Ministry of Industry and Information Technology Key Laboratory of Special Function and Smart Polymer Materials, Key Laboratory of Materials Physics and Chemistry of Ministry of Education for Extraordinary Conditions, Northwestern Polytechnical University, Xi'an 710072, P. R. China

[2] Spallation Neutron Source Science Center Institution, Dongguan 523803, P. R. China

[3] Institute of High Energy Physics, Chinese Academy of Sciences, Beijing 100049, P. R. China

[4] China Aerospace Science and Industry Corporation Sixth Academy, Hohhot 022185, P. R. China

[5] Xi'an Aerospace Composites Research Institute, Xi'an 710025, P. R. China

[6] China Aerospace Science and Technology Corporation, Beijing 100037, P. R. China

*Corresponding authors. E-mail: m_xiao_yana@nwpu.edu.cn (Xiaoyan Ma); houxiaoht@163.com (Xiao Hou)

S1 Characterization

Morphology Characterization. The morphology and element distribution of aerogel were analyzed by SEM (TESCAN, CLARA), TEM (FEI Talos F200X) and EDS (Oxford, INCA Xplore 30). The surface morphology and roughness of aerogel were analyzed by laser optical surface profiler (Bruker, Contour GT-K).

Porosity characteristics. The bulk density (ρ) of aerogels was obtained by calculating the geometric volume with a vernier caliper and measuring the weight with an analytical balance. Pore size distribution of aerogels was determined with a mercury intrusion porosimeter (Micromeritics, AutoPore IV 9500). The micropore of aerogel was analyzed by Small-angle X-ray scattering equipped with an 8 keV Cu $K\alpha$ 0.5 mm x 0.5 mm microfocus spot (30 W generator – 50 kV/0.6 mA) to provide large enough q space. The detector was positioned 50, 200, 400, 4000 mm away from the sample, to cover an angular range (0.01-1.00 Å^{-1} in scattering vector Q values). The precise sample-to-detector distance was determined with a silver behenate standard. Powder samples were held and measured within 2 Kapton film inside the instrument's sample holder, together with a blank without holding any samples for subtracting the background scattering. All experimental data refinements were done using the non-linear curve fit functionality, using Levenberg-Marquardt algorithm.

Chemical Structure. The chemical structure of precursors was characterized by FTIR (Bruker, Tensor 27) with the wavelength at the range of 400 to 4000 cm^{-1}. The chemical structures of aerogels were analyzed by XPS (Kratos, AXIS ULTRA DLD) with Al $K\alpha$ ray, 1486.6 eV energy, and 150 W power. All spectra were charge referenced against the C 1s peak at 284.8 eV to correct for charging effects during acquisition.

Ceramic/carbon structure. The ceramic/carbon structure of volumetric ablated aerogel was characterized by Raman spectra (WITec, Alpha300R), X-ray diffraction (Rigaku SmartLab)

with Ni-filtered Cu Kα radiation (40 kV, 30 mA, λ = 1.5418 Å) collected from 5 to 120° in 2θ with the step width of 0.01°.

Thermal stability properties. The TGA was carried out from the ambient temperature to 1000 °C using TA instruments (TGAQ50) at 20 °C/min under N_2. The TG-IR analysis was performed on the ThermoFisher (is50) and NETZSCH (STA449F3) under Ar atmosphere at the heating rate of 10 °C/min from 30 to 1000 °C, and the FT-IR resolution is 4 cm^{-1}.

Thermal insulation properties. Thermal conductivity (λ) and thermal diffusion (α) values of the aerogels were acquired through thermal constant analyze equipment Hot Disk (TPS2200), with the atmospheric pressure under the room temperature and a heating power of 5 mW for 10 s. The thermal images were recorded by a thermal infrared imager Guide sensmart (PS610). The ablative insulating behaviors were tested on a butane flame torch system for 60 s.

Finite Element Analysis of Ablation Behavior. The ablation model was calculated using the COMSOL Multiphysics software. The thermal response of the aerogel can be solved using the partial differential equation (PDE) established by the system. A 2D axisymmetric geometry model is created with dimensions φ30×10 mm^3, ablation time 60s, the natural cooling 40s, and the ablation temperature 1300 °C. The boundary conditions including heat flux and gas pressure are shown in Fig. S1. For heat flux, the surface boundary of the model is set to "Neumann boundary conditions" for calculating the surface energy changes, while the other boundaries are set to convective dissipation. The initial temperature is set to 303.15 K. For gas pressures, all boundaries are set to "Dirichlet boundary conditions" with an initial static pressure of 1 atm. The domain probe was inserted to output the temperature change parameter of the point with time to achieve the solution of the back temperature. The fully coupled transient solver is used for numerical calculation of the ablation model.

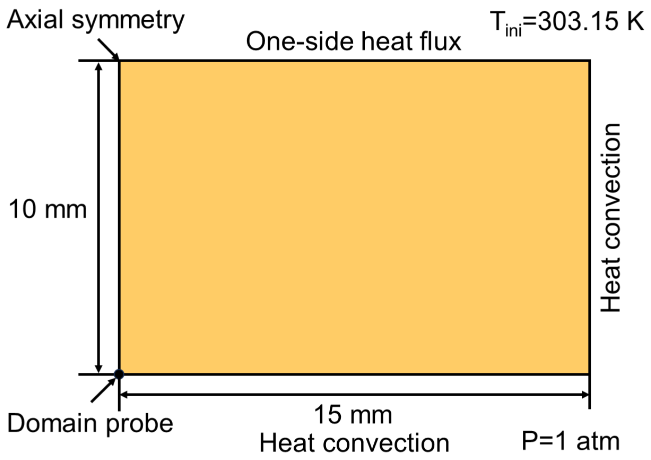

Fig. S1 The boundary condition of heat flux

Contact angles measurements. Water contact angles (WCA) were measured and calculated using a contact angle analyzer (Dataphysics OCA-15EC, Germany) using 2 μL water droplets.

Compression properties. Compression tests were measured on blocks by the universal test machine (SANS, CMT6303) with a displacement rate of 1 mm/min.

Electromagnetic Measurements. The EMI shielding performance of the samples was measured by a vector network analyzer (Anritsu, MS4644A) based on the wave-guide method in X-band (22.86 mm×10.16 mm).

Electrical conductivity. The electrical conductivities of the samples ablated at 800, 1000, 1200, 1400 and 1600 °C were obtained by four-point probing system (Guangzhou Four Probes, RTS-8). The electrical conductivity of samples ablated at 600 °C was tested by ultra-high resistance micro current insulation surface resistivity tester (Suzhou JingGe, ST2643). The conduction path diagram of LED lamp powered by electrochemical workstation with voltage of 1.5 V (CHI760E).

S2 Supplementary Figures and Tables

Chemical structure of hybrid phenolic precursors

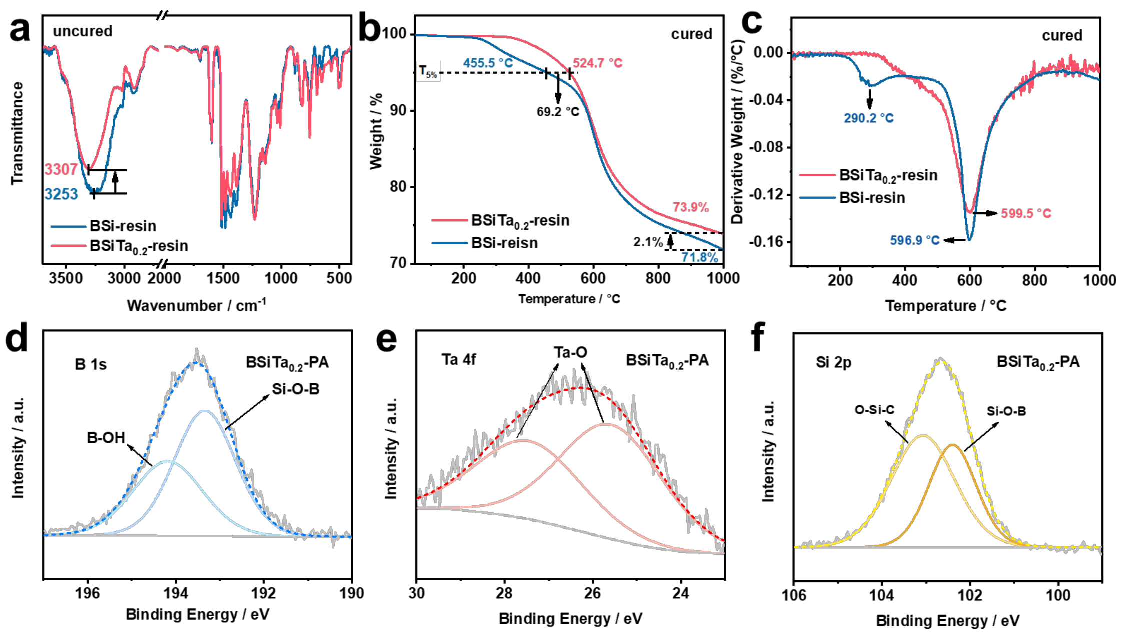

Fig. S2 The chemical structure of uncured hybrid resins: **a** the FT-IR of uncured BSi-resin and $BSiTa_{0.2}$-resin. The thermal stability of cured hybrid resins: **b-c** the TGA and DTG curves of cured BSi-resin and $BSiTa_{0.2}$-resin with curing process of 110 °C/1 h+130 °C/2 h+160 °C/2 h+180 °C/2 h+200 °C/1 h. The chemical structure of $BSiTa_{0.2}$-PA aerogels: **d-f** the XPS analysis of $BSiTa_{0.2}$-PA

Surface Morphology and Roughness of Aerogel

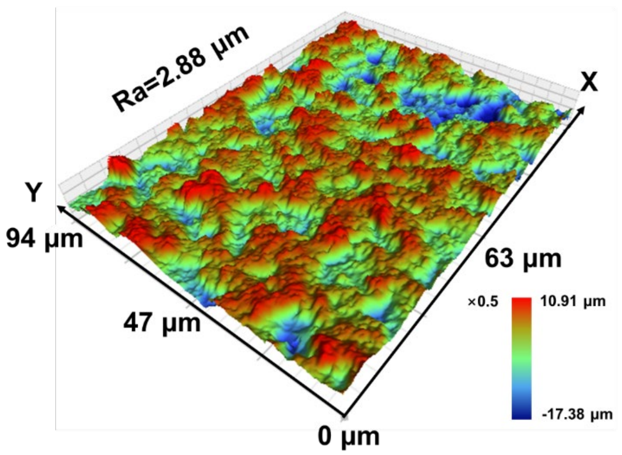

Fig. S3 Laser optical 3D surface image of the BSi-PA

Pore size distribution of the aerogels

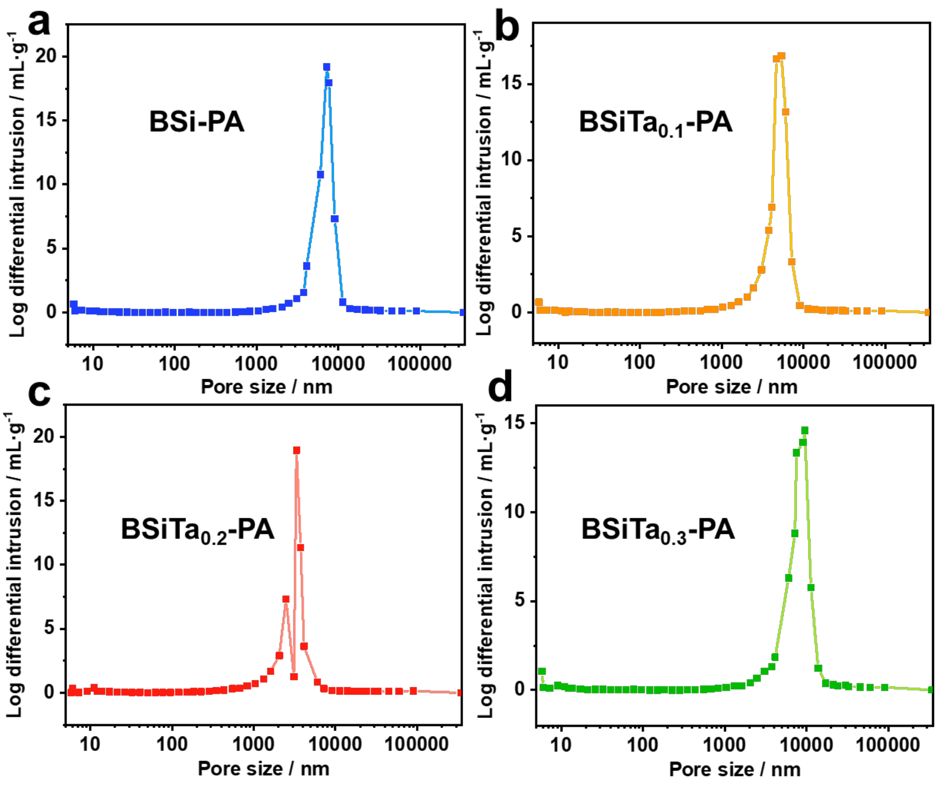

Fig. S4 Pore size distribution of the aerogels

Superhydrophobic properties

Hydrophobicity is also a key requirement for aerogels to be used outdoors [1]. The static water contact Angle (SCA) of aerogels with different tantalum content is greater than 150°, which indicates super-hydrophobicity, as shown in **Fig. S5**. When BSiTa$_{0.2}$-PA is immersed in water by an external force, the presence of an air cushion between the water and the aerogel results in reflectivity or a large number of microbubbles on the aerogel surface (**Fig. S6a-left**). This phenomenon indicates that the water is in the Cassie-Baxter state, and the interaction between the water and the aerogel is very weak. When different aqueous solutions (such as milk, orange juice, dye solution, sodium chloride solution, coffee and tea) are dropped on the surface of BSiTa$_{0.2}$-PA, the droplets stand stably on the aerogel in an almost spherical shape (**Fig. S6a-right**), and the SCA of these contaminative solutions is greater than 150°, as shown in **Fig. S6b**. This indicates that BSiTa$_{0.2}$-PA has broad-spectrum anti-pollution performance. The hydrophobicity of BSiTa-PA can be attributed to the following aspects: (1) The hydrophobic aromatic skeleton structure of the hybrid phenolic resin is stably chelated with the hydrolyzed product of tantalum precursor, resulting in a very low surface energy of the aerogel; (2) The multi-scale micro-nano structure of BSiTa-PA constructs the rough surface (**Fig. 2j**).

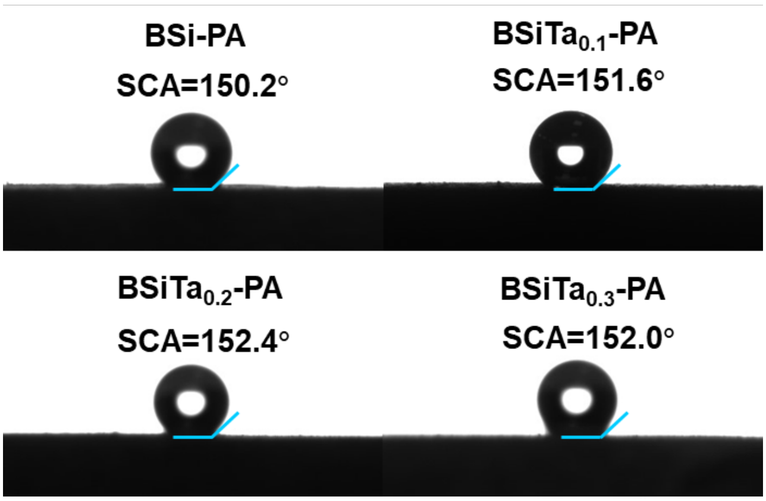

Fig. S5 Static water contact angles of different aerogels

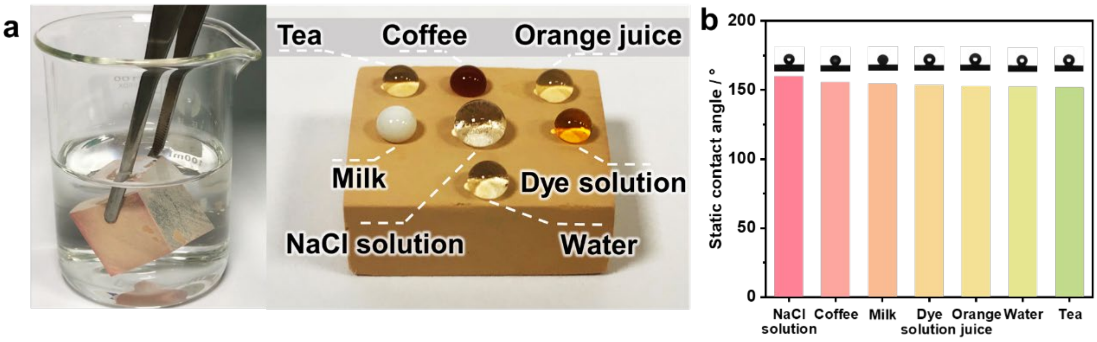

Fig. S6 Superhydrophobic properties. **a** Photograph of the hydrophobicity of the $BSiTa_{0.2}$-PA to different liquid stains. **b** Static contact angle of the different liquid on the surface of $BSiTa_{0.2}$-PA

Thermal insulation, infrared stealth performances and ablation devices

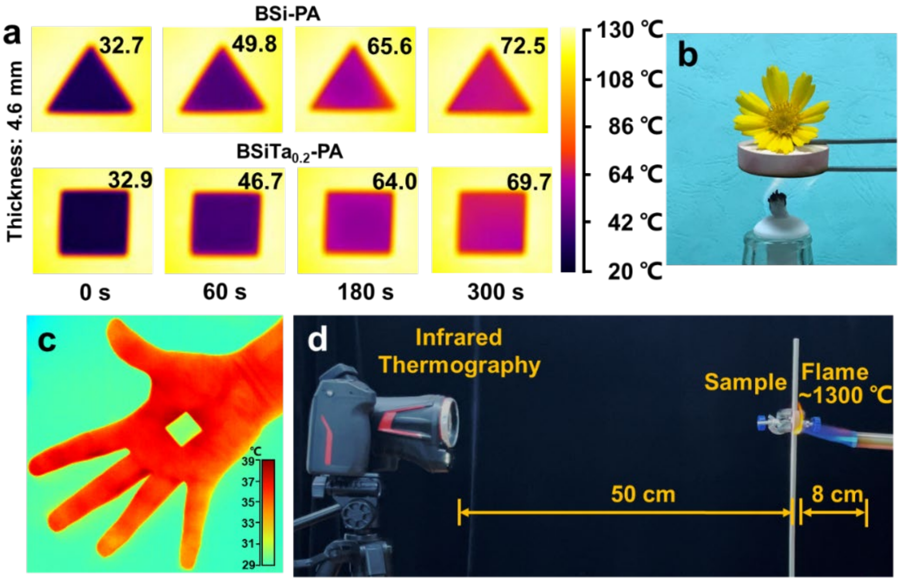

Fig. S7 **a** Static thermal insulation properties of BSi-PA and $BSiTa_{0.2}$-PA, **b** excellent thermal insulation performance of intuitive vision display, **c** infrared stealth property, **d** infrared thermal imager and butane torch flame ablation devices

Finite element method simulation of ablation process

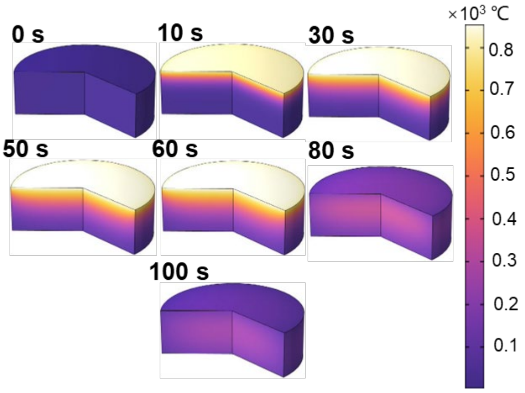

Fig. S8 Temperature field simulation cloud image of BSi-PA (0-60 s for ablation, 60-100 s for natural cooling)

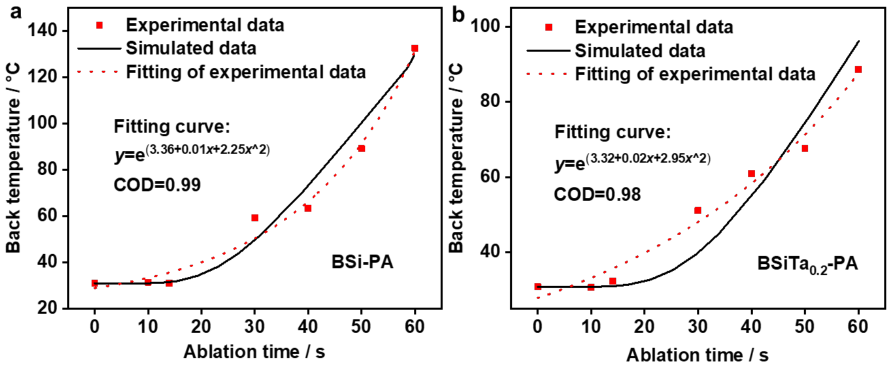

Fig. S9 Back temperature experimental and simulated results: **a** BSi-PA, **b** $BSiTa_{0.2}$-PA

Ceramic/carbon/pore structure transformation of volumetric ablated $BSiTa_{0.2}$-PA

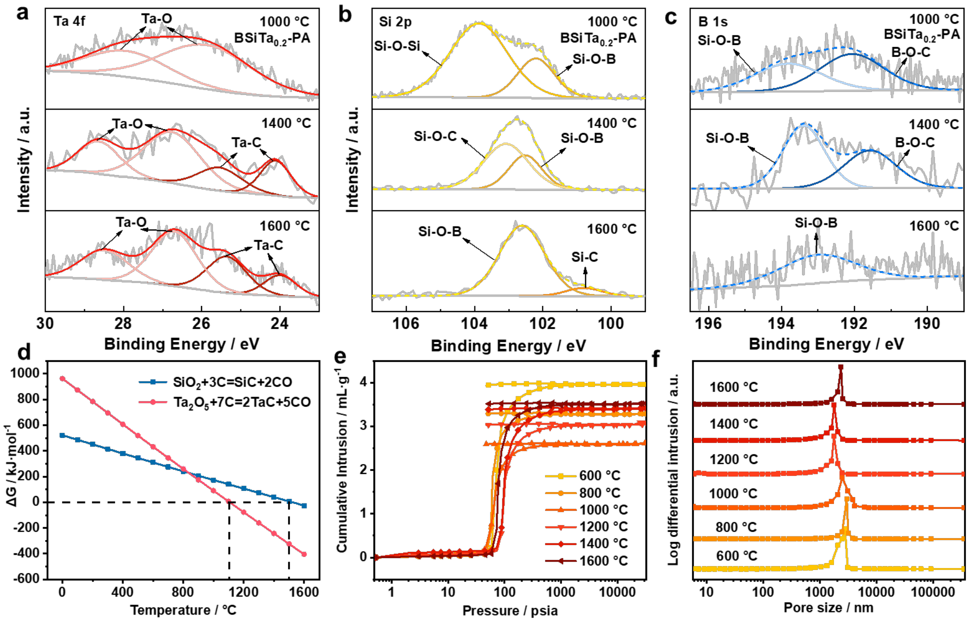

Fig. S10 Ceramic structure transformation: **a-c** XPS analysis of volumetric ablated $BSiTa_{0.2}$-PA at 1000, 1400, 1600 °C, **d** Gibbs free energy with temperature for ceramic reactions. Pore structure transformation: **e-f** MIP results of ablated $BSiTa_{0.2}$-PA

Model of SAXS fitting

For SAXS measurements, powder samples were held in place by two tape sheets, in which an empty cavity with two tape sheets was measured as a blank for the sample holder scattering signal. The scattering from the sample holder, when used, has been subtracted. The SAXS patterns were shown in log-log scale.

According to the theoretical models proposed by Saurel et al. [2], the SAXS signal in a range from 0.01 to 1.00 $Å^{-1}$ of microporous carbon powders is mainly contributed by two components: (i) a scattering signal I_{Porod} at a low Q range based on Porod's law (**Eq. S1**), ascribed to the macroscopic surface are of the powder grains; (ii) a signal I_{mp} in the intermediate Q range caused by microporosity whose profile depends on its nature (**Eq. S2**).

$$I_{Porod} = k \cdot Q^n + m \tag{S1}$$

where n is the Porod's final slope. A slope of -4 represents a smooth interface between domains in a multiphase system, while slopes between -3 and -4 characterize rough interface. In this work, the slopes are in the range from -3.6 to -3.9.

$$I_{mp} = a\frac{1}{1+C_1Q^2+C_2Q^4} \tag{S2}$$

where a, C_1 and C_2 are adjustable parameters. C_1 and C_2 can be extracted from the refinement fitting results to calculate the average pore-pore distance (d) and amphiphilic factor (f_a) considered as a disorder parameter as defined in the following **Eqs. S3 and S4**:

$$d = 2\pi/\sqrt{\left(\frac{1}{2\sqrt{C_1}} - \frac{C_1}{4C_2}\right)} \tag{S3}$$

$$f_a = \frac{C_1}{2\sqrt{C_2}} \tag{S4}$$

The average pore radius r can be estimated by analogy with the globulus form factor defined in **Eq. S5**:

$$r = \sqrt{5C_1} \tag{S5}$$

The SAXS fitting results listed as below.

Table S1 The fitting parameters (C_1 and C_2), and calculated d, f_a and r values for $BSiTa_{0.2}$-PA samples at pyrolysis temperature from 800 to 1600 °C

Sample	C_1	C_2	d / Å	f_a	r / Å
800 °C	14.93698±0.52364	106.13068±2.50875	54.38197	0.72496	8.64204
1000 °C	10.23262±0.45710	200.89142±3.58248	41.84813	0.36097	7.15284
1200 °C	9.62160±0.18376	210.96166±2.50460	41.40989	0.33122	6.93600
1400 °C	8.06900±0.47367	325.17117±6.52100	42.82709	0.22374	6.35181
1600 °C	4.28820±0.33562	788.50701±9.50635	48.99415	0.07636	4.63048

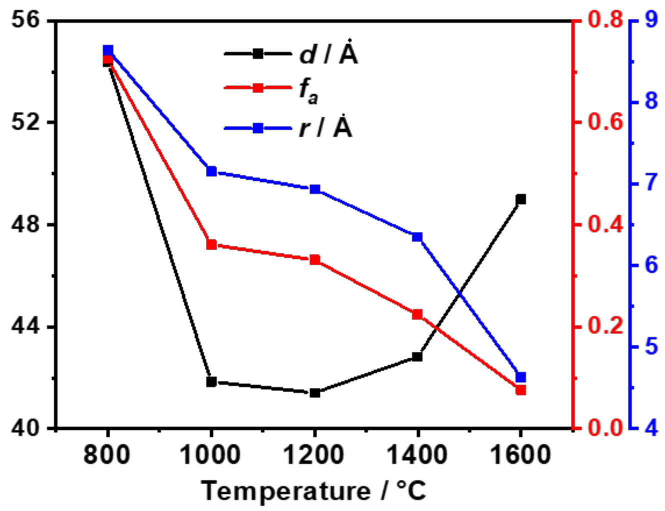

Fig. S11 The fitting results of SAXS (*d*: pore-pore distance; f_a: disorder parameter; *r*: average pore radius)

SEM, EDS and SCA of ablated aerogels

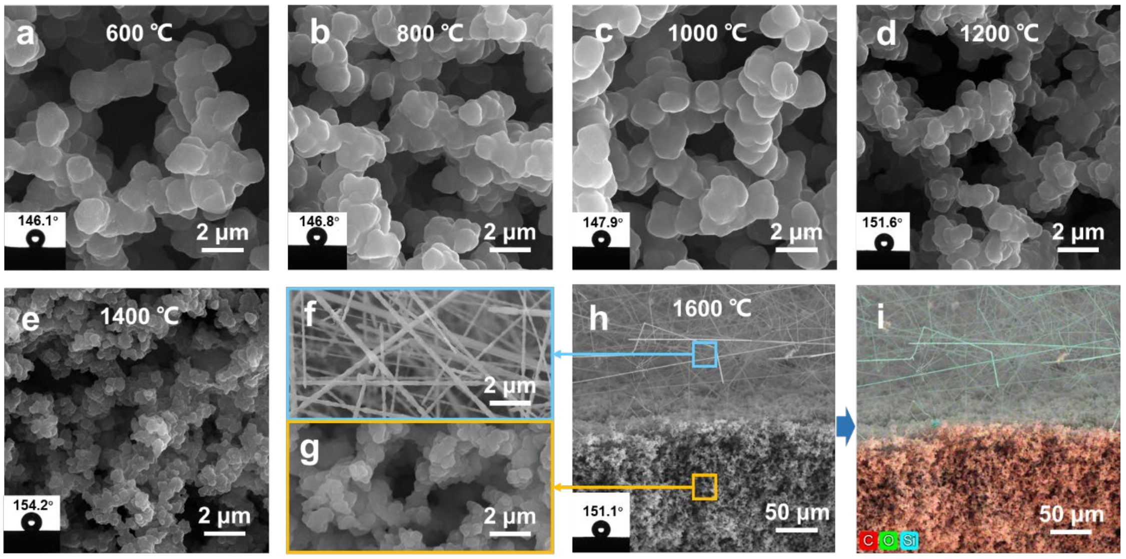

Fig. S12 Morphology and SCA of ablated $BSiTa_{0.2}$-PA at **a** 600 °C, **b** 800 °C, **c** 1000 °C, **d** 1200 °C, **d** 1400 °C, **f-h** 1600 °C, and **i** elemental mapping stack images of sample in 1600 °C

Density, pore, mechanical characteristic of the aerogels before and after volumetric ablation

Table S2 Density, pore size and mechanical characteristics of BSi-PA and BSiTa-PA

Sample	Density (g/cm^3)	Average macropore diameter (μm)	Specific surface area (m^2/g)	Compression modulus (MPa)	Specific modulus (kN·m/kg)
BSi-PA	0.180±0.006	6363.1	33.3	3.5±0.2	19.4±0.5
$BSiTa_{0.1}$-PA	0.179±0.007	4335.2	34.9	4.0±0.2	22.6±0.7
$BSiTa_{0.2}$-PA	0.180±0.003	2397.4	44.2	7.1±0.3	39.4±0.6
$BSiTa_{0.3}$-PA	0.179±0.002	6709.0	41.8	5.5±0.1	30.7±0.3

Table S3 Density, carbonization and mechanical properties of BSiTa$_{0.2}$-PA after volumetric ablation

Volume ablation temperature (°C)	Density (g/cm^3)	Char yield (%)	Liner shrinkage (%)	Average macropore diameter (nm)	Specific modulus (kN·m/kg)
600	0.176±0.006	62.9±0.6	14.0±0.5	1741.7	88.5±0.6
800	0.207±0.002	57.8±0.5	20.3±0.8	2328.9	100.9±0.3
1000	0.206±0.001	55.3±1.0	21.5±0.5	2161.5	128.8±0.8
1200	0.206±0.005	55.2±0.2	21.6±0.5	1315.6	209.2±0.7
1400	0.196±0.007	52.4±0.3	21.9±0.8	1291.6	272.8±0.5
1600	0.195±0.003	52.2±0.5	22.2±1.2	1742.9	151.5±1.0

EMI shielding performance of the aerogels volumetric ablated at different temperatures

Table S4 The EM reflection, EM absorption and EM SE of BSiTa$_{0.2}$-PA at different volumetric ablation temperatures

Volumetric ablation temperature (°C)	EM reflection (dB)	EM absorption (dB)	EM SE (dB)
600	0.076	0.068	0.14
800	19.02	6.38	25.4
1000	19.68	9.23	28.9
1200	19.38	9.58	29.0
1400	20.73	10.86	31.6
1600	18.66	9.93	28.6

Comparisons of performances

Comparison with other types of high-performance aerogels is shown in Table S4. The preparation efficiency of BSiTa-PA developed in this work is significantly higher and the preparation cost is lower, which is very conducive to the further application development. Moreover, BSiTa-PA possesses significant advantages in thermal stability and shape-maintaining ability, which provides an important guarantee for the improvement of its ablative resistance and electromagnetic shielding performance.

Table S5 Comparisons of performances of BSiTa-PA with other high-performance aerogels

Materials	Preparation duration	Drying method	Thermal stability	Liner shrinkage	Refs.
LigSi-3	More than 110 h	Freeze-dried	54.8% at 800 °C (N_2, unspecified)	-	[S1]
PBDAZ-100	More than 78 h	Supercritical fluid CO_2	40% char yield at 800 °C (Ar, 2.5 °C/min)	-	[S3]
PBDAZ-240	More than 102 h		61% char yield at 800 °C (Ar, 2.5 °C/min)	18.0%<br>600 °C: 24.4%<br>800 °C: 29.0%	
PCP	34-82 h	Ambient pressure drying with solvent exchange	~48% at 800 °C (N_2, 10 °C/min)	3.2%	[S4]
BC-Si3	More than 50 h	Supercritical fluid CO_2 with solvent exchange	~10% at 800 °C (N_2, 10 °C/min)	-	[S5]
CNF@Al-MIL-53	More than 120 h	Freeze-dried	~15% at 750 °C (N_2, 10 °C/min)	-	[S6]
PANF aerogel	-	Freeze-dried	~50% at 800 °C (N_2, 10 °C/min)	50.2-76.1%	[S7]

A-NC<br>T-NC<br>M-NC	More than 50 h	Freeze-dried	5~25% at 600 °C (N_2, 20 °C/min)	-	[S8]
BSiTa$_{0.2}$-PA	Less than 32 h	Ambient pressure drying without solvent exchange	61.2% char yield at 800°C (N_2, 20 °C/min)	3.0±0.3%<br>600°C: 14.0±0.5%<br>800°C: 20.3±0.8%	This work

Equations

According to the Young-Laplace equation, the capillary pressure (*P*) could be calculated as [S9]:

$$P = -2\gamma cos\theta/r \tag{S1}$$

where γ is the solvent surface tension, θ the contact angle between the solvent and the pore wall, and *r* the pore diameter.

The λ_{solid} can be expressed as [S10]:

$$\lambda_{\text{solid}} = \frac{1}{3}\int c_{\text{v}}\rho v_{\text{ph}} l_{\text{ph}} \tag{S2}$$

where c_{v}, ρ, v_{ph}, and l_{ph} are the specific heat capacity, material density, mean velocity, and mean free path of phonons, respectively. It can be seen that the λ_{solid} decreases as the density decreases.

Supplementary References

[S1]Q. Fan, R. Ou, X. Hao, Q. Deng, Z. Liu, L. Sun, C. Zhang, C. Guo, X. Bai, Q. Wang. Water-induced self-assembly and in situ mineralization within plant phenolic glycol-gel toward ultrastrong and multifunctional thermal insulating aerogels. ACS Nano **16**(6), 9062-9076 (2022). https://doi.org/10.1021/acsnano.2c00755

[S2]D. Saurel, J. Segalini, M. Jauregui, A. Pendashteh, B. Daffos, P. Simon, M. Casas-Cabanas. A saxs outlook on disordered carbonaceous materials for electrochemical energy storage. Energy Storage Mater. **21**, 162-173 (2019). https://doi.org/10.1016/j.ensm.2019.05.007

[S3]V. A. Edlabadkar, S. Gorla, R. U. Soni, A. B. M. S. u. Doulah, J. Gloriod, S. Hackett, N. Leventis, C. Sotiriou-Leventis. Polybenzodiazine aerogels: All-nitrogen analogues of polybenzoxazines—synthesis, characterization, and high-yield conversion to nanoporous carbons. Chem. Mater. (2023). https://doi.org/10.1021/acs.chemmater.2c02797

[S4]J. Li, P. Guo, C. Hu, S. Pang, J. Ma, R. Zhao, S. Tang, H.-M. Cheng. Fabrication of large aerogel-like carbon/carbon composites with excellent load-bearing capacity and thermal-insulating performance at 1800 °C. ACS Nano **16**(4), 6565-6577 (2022). https://doi.org/10.1021/acsnano.2c00943

[S5]J. Zhang, Y. Cheng, M. Tebyetekerwa, S. Meng, M. Zhu, Y. Lu. "Stiff–soft" binary synergistic aerogels with superflexibility and high thermal insulation performance. Adv. Funct. Mater. **29**(15), 1806407 (2019). https://doi.org/10.1002/adfm.201806407

[S6]S. Zhou, V. Apostolopoulou-Kalkavoura, M. V. Tavares da Costa, L. Bergström, M. Strømme, C. Xu. Elastic aerogels of cellulose nanofibers@metal–organic frameworks for

thermal insulation and fire retardancy. Nano-Micro Lett. **12**(1), 9 (2019). https://doi.org/10.1007/s40820-019-0343-4

[S7] C. Xie, S. Liu, Q. Zhang, H. Ma, S. Yang, Z.-X. Guo, T. Qiu, X. Tuo. Macroscopic-scale preparation of aramid nanofiber aerogel by modified freezing–drying method. ACS Nano. **15**(6), 10000-10009 (2021). https://doi.org/10.1021/acsnano.1c01551

[S8] S. Jiang, M. Zhang, W. Jiang, Q. Xu, J. Yu, L. Liu, L. Liu. Multiscale nanocelluloses hybrid aerogels for thermal insulation: The study on mechanical and thermal properties. Carbohydrate Polymers **247**(116701 (2020). https://doi.org/10.1016/j.carbpol.2020.116701

[S9] J. Zhang, J. Zheng, M. Gao, C. Xu, Y. Cheng, M. Zhu. Nacre-mimetic nanocomposite aerogels with exceptional mechanical performance for thermal superinsulation at extreme conditions. Adv. Mater. **35**(29), 2300813 (2023). https://doi.org/10.1002/adma.202300813

[S10] H. Huang, X. Yan, X. Jin, C. Wu, Y. Pan, H. Wang, C. Zhu, C. Hong, W. Han, X. Zhang. Flexible and interlocked quartz fibre reinforced dual polyimide network for high-temperature thermal protection. J. Mater. Chem. A **11**(18), 9931-9941 (2023). https://doi.org/10.1039/D3TA01413D
